# Immune checkpoint inhibitor-associated aseptic meningitis: a pharmacovigilance study using the FDA adverse event reporting system (2011–2024)

**DOI:** 10.3389/fimmu.2025.1654301

**Published:** 2025-10-03

**Authors:** Hui Guo, Yuzhu Pang, Qian Guo, Ze Wang, Haixiong Wang

**Affiliations:** ^1^ Department of Pharmacy, Shanxi Cardiovascular Hospital, Taiyuan, Shanxi, China; ^2^ School of Management, Shanxi Medical University, Taiyuan, Shanxi, China; ^3^ Department of Pharmacy, Second Hospital of Shanxi Medical University, Taiyuan, Shanxi, China

**Keywords:** immune checkpoint inhibitors, aseptic meningitis, Food and Drug Administration’s Adverse Event Reporting System, pharmacovigilance, data mining

## Abstract

**Objective:**

Immune checkpoint inhibitors (ICIs) are pivotal in oncology but carry risks of immune-related adverse events (irAEs). Aseptic meningitis (AM) represents a serious neurological irAE, yet real-world evidence on regimen-specific risk variations remains limited. This study aimed to characterize AM reporting patterns and safety signals across ICI regimens using FDA Adverse Event Reporting System (FAERS) data.

**Methods:**

We analyzed FAERS reports (January 2011–December 2024) for ICIs-associated AM. Descriptive statistics summarized demographics, clinical profiles, and temporal trends. Disproportionality analyses employed four algorithms: Reporting Odds Ratio (ROR), Proportional Reporting Ratio (PRR), Bayesian Confidence Propagation Neural Network (BCPNN), and Multi-item Gamma Poisson Shrinker (MGPS).

**Results:**

Among 498 ICIs-associated AM reports, monotherapy predominated (78.7%) with pembrolizumab (34.9%), ipilimumab/nivolumab (21.3%), nivolumab (17.1%), and atezolizumab (15.9%) as leading agents. Patients had a median age of 64 years; 98% met serious adverse event criteria. Hospitalization (45.8%) was the most common outcome. Symptom onset was rapid (median: 34 days). Disproportionality analysis revealed pronounced signals for ipilimumab/nivolumab (ROR 5.71, 95% CI 4.71–6.91) and ipilimumab monotherapy (ROR 4.21, 95% CI 3.05–5.82). Anti-PD-1 agents collectively showed moderate association (ROR 2.55, 95% CI 2.25–2.88).

**Conclusions:**

ICIs-associated AM presents a clinically significant safety concern, particularly with ipilimumab-containing regimens. Rapid symptom onset underscores the need for vigilant neurological monitoring during early treatment phases. These findings warrant integration into clinical risk-assessment protocols and warrant further mechanistic investigation.

## 1 Introduction

The advent of immune checkpoint inhibitors (ICIs) has revolutionized oncology since the 2011 approval of ipilimumab, the first-in-class cytotoxic T-cell–associated protein-4 (CTLA-4) inhibitor. These agents—including programmed cell death protein-1 (PD-1)/programmed death-ligand 1 (PD-L1) inhibitors and next-generation checkpoint modulators—have transformed therapeutic paradigms for advanced malignancies, establishing ICIs as a cornerstone of precision cancer therapy (1). Despite their remarkable antitumor efficacy, their expanding use has unveiled a spectrum of immune-related adverse events (irAEs) affecting multiple organ systems, with gastrointestinal, endocrine, dermatologic, hepatic, pulmonary, and articular manifestations being most prevalent. While monotherapy typically results in severe irAEs in approximately 13% of cases (2), fatal complications occur in 0.3–1.3% of treated patients (3), underscoring the critical need for improved management strategies.

Although rarely, ICI therapy can lead to Immune-related neurologic adverse events (irNAEs), including myositis, myasthenic syndromes, cranial neuropathies, encephalitis, meningitis (4). irNAEs occur early into the treatment with ICIs. Of those, irNAEs are rare and may present as unspecific symptoms including headache, vomiting or dizziness. More severe presentations comprise polyradiculitis, myasthenia gravis, encephalitis or demyelinating disease (5, 6). A meta-analysis by Cuzzubbo et al. demonstrated distinct irNAEs risks across ICI classes, with anti-CTLA4 antibodies associated with the lowest incidence (3.8%), followed by PD-1 inhibitors (6.1%), and combination therapy showing the highest risk (12.0%) (5). Previous research has suggested that irNAEs have been implicated in nearly half of all deaths associated with ICIs (3). Meningitis triggered by ICIs is overall rare (3% of all n-irAEs) (7), but probably under-reported. This comparatively low neurotoxicity incidence may reflect anatomical protections including the blood-brain barrier, blood-nerve barrier, and unique immunomodulatory properties of central nerves system-resident microglia within the tumor microenvironment.

In particular, ICIs-induced aseptic meningitis (AM) is a rare but clinically significant neurological irAE. Although less fatal than immune-related myasthenia gravis, AM can still lead to substantial morbidity and requires timely recognition and management (8). The European Society for Medical Oncology (ESMO) recommends systematic diagnostic evaluation, including MRI, lumbar puncture, viral screening, and serological analysis for suspected cases (9). When meningeal irAEs are clinically significant, management typically features high-dose steroid administration for at least 4 to 8 weeks with decreasing doses (9). A case series by Nannini et al. reported melanoma as the predominant malignancy (52.5%), dual ICI therapy in 40% of cases, and symptom onset occurring at a median of two treatment cycles (10). Notably, while corticosteroids resolved symptoms in 87.5% of patients, therapy rechallenge carried substantial recurrence risks—particularly with the original regimen—highlighting the need for individualized management. Nevertheless, population—level evidence on ICI—induced AM remains scarce. To address this gap, we conducted a pharmacovigilance analysis using the FDA Adverse Event Reporting System (FAERS), aiming to: (1) characterize clinical manifestations and outcomes, and (2) assess potential safety signals between different ICIs and AM.

## 2 Methods

### 2.1 Data source

This retrospective pharmacovigilance study utilized individual case safety reports (ICSRs) from the FDA Adverse Event Reporting System (FAERS), a spontaneous surveillance database central to post-marketing drug safety monitoring. FAERS archives adverse events, medication errors, and product quality complaints, capturing demographic, drug, indication, outcome, reaction, and therapeutic data. FAERS constitutes an integral component of the FDA’s post-approval safety surveillance infrastructure for pharmaceuticals and biologics, functioning within an established spontaneous reporting framework. The repository comprehensively documents standardized pharmacovigilance data elements encompassing demographic variables, drug exposure details, therapeutic indications, clinical outcomes, adverse reaction profiles, reporter qualifications, and concomitant interventions.

### 2.2 Data mining

We extracted ICSRs where ICIs were designated as suspected agents (January 2011–December 2024). Cases of AM were identified using Medical Dictionary for Regulatory Activities (MedDRA v26.0) Standardized MedDRA Query (SMQ) code 20000134 and clinically synonymous terms. To ensure data integrity, duplicate records sharing ≥3 identical fields (event date, age, sex, reporter country) were removed and cases with ICIs initiation dates after meningitis onset were eliminated.

### 2.3 Statistical analysis

The clinical characteristics, such as age, sex, primary data source, outcomes, reported year, source region, and indication, were stratified by ICI class. To detect potential safety signals between ICIs and AM, we applied disproportionality analysis (reporting odds ratio, ROR; proportional reporting ratio, PRR) and Bayesian methodologies (Bayesian Confidence Propagation Neural Network, BCPNN; Multi-item Gamma Poisson Shrinker, MGPS). Algorithm-specific thresholds (**Table 1** (11)) defined signal positive, with concurrent fulfillment in ≥2 methods required to establish a validated association. All analyses were implemented in SPSS 26.0 (IBM Corp., Armonk, NY).

**Table 1:** Summary of major algorithms used for signal detection.

Algorithms	Equation	Criteria
ROR	ROR=ad/bc 95%CI=e^ln(ROR)^ ^±|^ ^1.96(1/a+1/b+1/c+1/d)^0.5^	95%CI>1, N≥2
PRR	PRR = a(c + d)/c/(a + b) χ^2^ = [(ad−bc)^2](a + b + c + d)/[(a + b) (c + d)(a + c)(b + d)]	PRR≥2, χ^2^≥4, N≥3
BCPNN	IC = log_2_^a(a + b + c + d)(a + c)(a + b)^ IC025=e^ln(IC)-1.96(1/a+1/b+1/c+1/d)^0.5^	IC025>0
MGPS	EBGM = a(a + b + c + d)/(a + c)/(a + b) EBGM05=e^ln(EBGM)-1.64(1/a+1/b+1/c+1/d)^0.5^	EBGM05>2, N>0

a, the number of reports with suspect adverse drug event (ADE) of the suspect drug; b, the number of reports with all other ADEs of the suspect drug; c, the number of reports with the suspect ADE of all other drugs; d, the number of reports with all other ADEs of all other drugs; ROR, reporting odds ratio; CI, confidence interval; N, the number of co-occurrences; PRR, proportional reporting ratio; χ^2^, chi-squared; BCPNN, Bayesian confidence propagation neural network; IC, information component; IC025, the lower limit of the 95% two-sided CI of the IC; MGPS, multi-item gamma Poisson shrinker; EBGM, empirical Bayesian geometric mean; EBGM05, the lower 95% one-sided CI of EBGM.

## 3 Results

### 3.1 Descriptive analysis from FAERS

**Figure 1:**
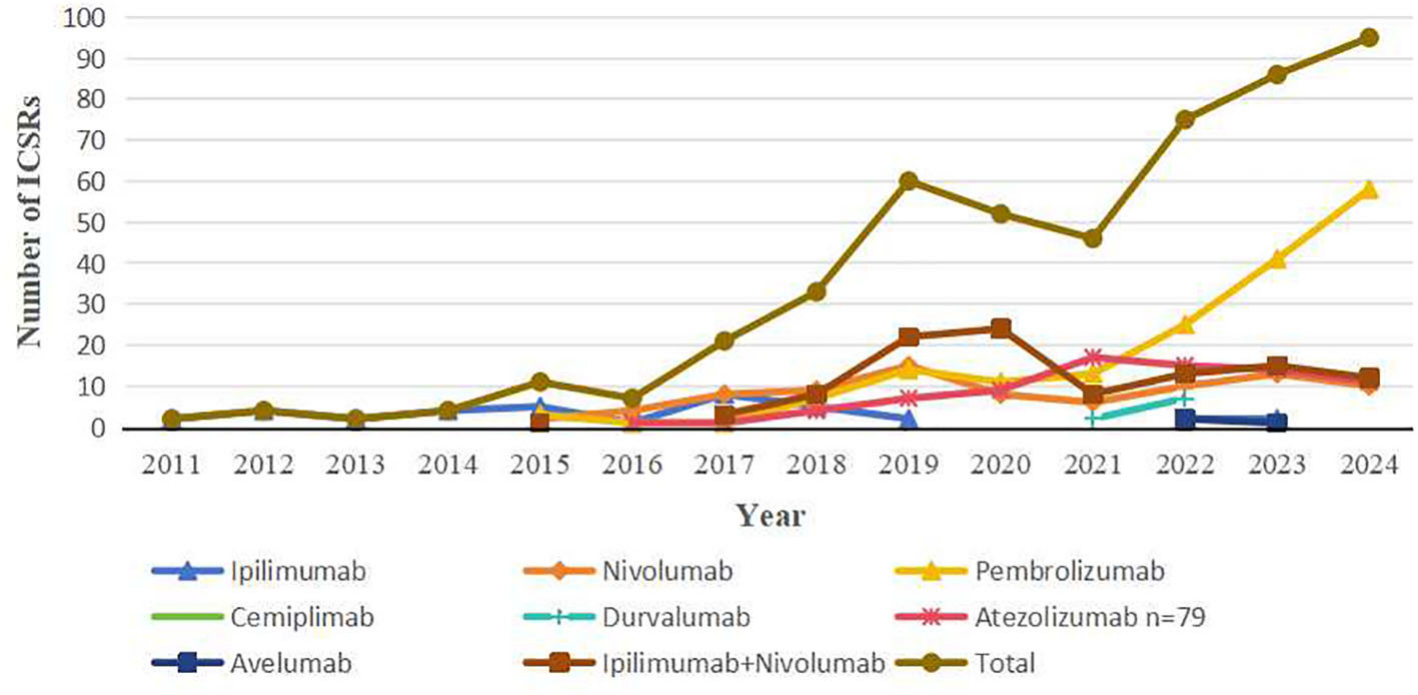
Aseptic meningitis related Individual Safety Reports induced by ICIs (2022).

From January 2011 to December 2024, FAERS documented 498 ICIs-associated AM reports. Monotherapy accounted for 78.7% (n=392) of cases, with pembrolizumab (34.9%, n=174), ipilimumab/nivolumab combination (21.3%, n=106), nivolumab (17.1%, n=85), and atezolizumab (15.9%, n=79) constituting the predominant agents. Less frequent associations included ipilimumab (7.4%, n=37), durvalumab (1.8%, n=9), cemiplimab (1.0%, n=5), and avelumab (0.6%, n=3). ICSRs of other ICI regimens were not discovered. Demographic/clinical profiles are detailed in **Table 2**. Among 393 patients (median age: 64 years; range: 20–90), males accounted for 230 (46.2%) and females 207 (41.6%), with sex not reported in 61 cases (12.2%). Notably, 98% met FDA serious adverse event criteria. Reports originated primarily from Asia (44.2%), the Americas (37.3%), and Europe (16.7%), with healthcare professionals submitting 94.6%. Hospitalization was the most common outcome (45.8%), followed by other serious events (32.1%), death (11.4%), and life-threatening status (6.4%). Head/neck cancers (24.7%) and haematopoietic and lymphoid tissues (16.1%) represented leading indications. Temporal analysis revealed a marked increase in cases—from 2 (2011) to 95 (2024)—peaking in 2025 (**Figure 1**), paralleling expanded ICIs utilization. Symptom onset occurred rapidly (median: 34 days; range: 0-2194; n=225), with 71.6% emerging within three months of therapy initiation. The median time to onset of atezolizumab was 10 days (range: 0-730). Notably, the ipilimumab/nivolumab combination therapy demonstrated a shorter median time to onset than either ipilimumab (32 vs. 36.5 days) or nivolumab monotherapy (32 vs. 81 days).

**Table 2:** Clinical characteristics of patients with ICI-associated aseptic meningitis collected from the FAERS database (January 2011 to December 2024).

Variables	Ipilimumab n=37	Nivolumab n=85	Pembrolizumab n=174	Cemiplimab n=5	Durvalumab n=9	Atezolizumab n=79	Avelumab n=3	Ipilimumab+nivolumab n=106	Total n=498
Age median (range)	53 (27-78) n=19	64.5 (27-90) n=60	67 (21-86) n=145	61 (48-78) n=3	69 (69-69) n=7	68 (30-90) n=66	83.5 (81-86) n=2	60 (20-90) n=91	64 (20-90) n=393
Sex									
Male	13 (35.1)	42 (49.4)	85 (48.9)	–	1 (11.1)	32 (40.5)	1 (33.3)	56 (52.8)	230 (46.2)
Female	13 (35.1)	31 (36.5)	80 (46.0)	5 (100.0)	7 (77.8)	35 (44.3)	1 (33.3)	35 (33.0)	207 (41.6)
Not reported	11 (29.7)	12 (14.1)	9 (5.2)	–	1 (11.1)	12 (15.2)	1 (33.3)	15 (14.2)	61 (12.2)
Primary source									
Healthcare professional	33 (89.2)	82 (96.5)	163 (93.7)	3 (60.0)	8 (88.9)	77 (97.5)	3 (100.0)	102 (96.2)	471 (94.6)
Consumer	4 (10.8)	3 (3.5)	11 (6.3)	2 (40.0)	1 (11.1)	2 (2.5)	–	4 (3.8)	27 (5.4)
Outcomes									
Non-serious	–	1 (1.2)	7 (4.0)	1 (20.0)	–	–	–	1 (0.9)	10 (2.0)
Hospitalization	18 (48.6)	44 (51.8)	88 (50.6)	1 (20.0)	6 (66.7)	25 (31.6)	1 (33.3)	45 (42.5)	228 (45.8)
Disability	1 (2.7)	3 (3.5)	5 (2.9)	–	–	2 (2.5)	–	–	11 (2.2)
Life-threatening	2 (5.4)	3 (3.5)	11 (6.3)	–	–	4 (5.1)	–	12 (11.3)	32 (6.4)
Death	4 (10.8)	7 (8.2)	16 (9.2)	1 (20.0)	–	18 (22.8)	1 (33.3)	10 (9.4)	57 (11.4)
Other	12 (32.4)	27 (31.8)	47 (27.0)	2 (40.0)	3 (33.3)	30 (38.0)	1 (33.3)	38 (35.8)	160 (32.1)
Source region									
Africa	–	–	–	–	–	3 (3.8)	–	–	3 (0.6)
Asia	2 (5.4)	29 (34.1)	82 (47.1)	2 (40.0)	2 (22.2)	59 (74.7)	–	44 (41.5)	220 (44.2)
Europe	16 (43.2)	38 (44.7)	64 (36.8)	1 (20.0)	6 (66.7)	11 (13.9)	2 (66.7)	48 (45.3)	186 (37.3)
North America	18 (48.6)	18 (21.2)	26 (14.9)	2 (40.0)	1 (11.1)	5 (6.3)	1 (33.3)	12 (11.3)	83 (16.7)
Oceania	1 (2.7)	–	1 (0.6)	–	–	–	–	–	2 (0.4)
South America	-	–	1 (0.6)	–	–	1 (1.3)	–	1 (0.9)	3 (0.6)
Indication									
Bone tumor	–	–	3 (1.7)	–	–	23 (29.1)	–	2 (1.9)	28 (5.6)
Breast cancer	–	–	20 (11.5)	2 (40.0)	–	1 (1.3)	–	–	23 (4.6)
Gastrointestinal cancer	–	4 (4.7)	1 (0.6)		–	–	1 (33.3)	–	6 (1.2)
Haematopoietic and lymphoid tissues	–	12 (14.1)	5 (2.9)	1 (20.0)	–	3 (3.8)	1 (33.3)	58 (54.7)	80 (16.1)
Head and neck cancer	–	3 (3.5)	63 (36.2)	1 (20.0)	9 (100.0)	42 (53.2)	–	5 (4.7)	123 (24.7)
Mesothelioma	–	–	27 (15.5)	–	–	3 (3.8)	1 (33.3)	29 (27.4)	60 (12.0)
Neuroendocrine neoplasm	–	–	31 (17.8)	–	–	–	–	–	31 (6.2)
Skin cancer	24 (64.9)	22 (25.9)	1 (0.6)	–	–	–	–	–	47 (9.4)
Tumors of female reproductive organs	–	–	1 (0.6)	–	–	–	–	3 (2.8)	4 (0.8)
Tumors of respiratory system	–	26 (30.6)	–	–	–	–	–	2 (1.9)	28 (5.6)
Tumors of urinary system	4 (10.8)	11 (12.9)	1 (0.6)	–	–	–	–	–	16 (3.2)
Unknown or missing	9 (24.3)	7 (8.2)	21 (12.1)	1 (20.0)	–	7 (8.9)	–	7 (6.6)	52 (10.4)
Time to onset, days	36.5 (9-540) n=12	81 (0-2194) n=33	43 (0-1241) n=92	52 (24-92) n=3	7.5 (1-14) n=2	10 (0-730) n=38	14 (14-14) n=1	32 (0-336) n=46	34 (0-2194) n=225

### 3.2 Signal values associated with different ICIs

As delineated in **Table 3**, multiple ICI regimens demonstrated significant safety signals for AM. The strongest association emerged with ipilimumab/nivolumab combination therapy (ROR 5.71, 95% CI 4.71–6.91; IC025 47.81), exceeding the signal magnitude of all monotherapies. Among monotherapies, ipilimumab exhibited the highest disproportionality (ROR 4.21, 95% CI 3.05–5.82), followed by atezolizumab (ROR 3.40, 95% CI 2.72–4.24) and pembrolizumab (ROR 2.77, 95% CI 2.38–3.21). Notably, anti-PD-1 agents collectively showed significant but moderate association (ROR 2.55, 95% CI 2.25–2.88). Statistical consistency was observed across all four algorithms for ipilimumab/nivolumab, ipilimumab, pembrolizumab and atezolizumab. Exceptions included durvalumab (ROR 1.12, 95% CI 0.58–2.15) and avelumab (ROR 1.31, 95% CI 0.42–4.06), where confidence intervals crossed unity, indicating no significant signal. Direct comparison revealed that ipilimumab/nivolumab combination carried 35% higher reporting odds than nivolumab monotherapy (ROR 2.64, 95% CI 1.98–3.51).

**Table 3:** Associations of different ICI regimens with aseptic meningitis.

ICI regimens	N	ROR (95% CI)	PRR (χ^2^)	IC (IC025)	EBGM (EBGM05)
Total	498	3.04 (2.78, 3.32)	3.03 (656.68)	63.32 (57.90)	2.96 (2.74)
Ipilimumab	37	4.21 (3.05, 5.82)	4.19 (89.82)	55.31 (40.02)	4.18 (3.19)
Anti-PD-1	264	2.55 (2.25, 2.88)	2.54 (242.19)	61.71 (54.63)	2.51 (2.27)
Nivolumab	85	2.16 (1.74, 2.67)	2.15 (52.26)	58.67 (47.39)	2.15 (1.80)
Pembrolizumab	174	2.77 (2.38, 3.21)	2.76 (192.98)	60.39 (51.99)	2.74 (2.41)
Cemiplimab	5	3.00 (1.25, 7.23)	3.00 (6.65)	50.02 (20.78)	2.99 (1.44)
Anti-PD-L1	91	2.59 (2.10, 3.20)	2.58 (83.96)	58.48 (47.35)	2.57 (2.16)
Durvalumab	9	1.12 (0.58, 2.15)	1.12 (0.11)	53.13 (27.63)	1.12 (0.65)
Atezolizumab	79	3.40 (2.72, 4.24)	3.39 (132.38)	57.81 (46.32)	3.37 (2.80)
Avelumab	3	1.31 (0.42, 4.06)	1.31 (0.22)	49.74 (16.02)	1.31 (0.51)
Ipilimumab+ nivolumab	106	5.71 (4.71, 6.91)	5.68 (405.71)	57.91 (47.81)	5.64 (4.80)
Ipilimumab/ nivolumab vs. nivolumab		2.64 (1.98, 3.51)			

ICI, immune checkpoint inhibitor; N, the number of reports of ICI-associated aseptic meningitis; ROR, reporting odds ratio; CI, confidence interval; PRR, proportional reporting ratio; χ^2^, chi-squared; IC, information component; EBGM, empirical Bayes geometric mean.

**Table 4** delineates Preferred Term (PT)-level safety signals for noninfectious meningitis across ICI regimens. Ipilimumab, nivolumab, and pembrolizumab each demonstrated associations with five distinct PTs, indicating comparable signal breadth. In contrast, avelumab exhibited no significant signals while cemiplimab yielded only one detectable association. Meningitis and aseptic meningitis emerged as the predominant PTs across most regimens, with ipilimumab/nivolumab showing the most robust associations for these specific conditions.

**Table 4:** Signal strength for ICI based on PT level in FAERS.

PT	Ipilimumab		Nivolumab		Pembrolizumab		Cemiplimab		Durvalumab		Atezolizumab		Ipilimumab +nivolumab	
	N	ROR (95% CI)	N	ROR (95% CI)	N	ROR (95% CI)	N	ROR (95% CI)	N	ROR (95% CI)	N	ROR (95% CI)	N	ROR (95% CI)
Arachnoiditis	2	11.60 (2.89, 46.63)												
Meningeal disorder			2	4.86 (1.21, 19.63)	2	3.02 (0.75, 12.21)					2	8.24 (2.04, 33.25)		
Meningism	2	17.27 (4.29, 69.61)					2	91.65 (22.72, 369.60)			3	9.83 (3.14, 30.77)		
Meningitis	9	4.64 (2.41, 8.93)	41	4.76 (3.50, 6.48)	71	5.18 (4.09, 6.55)			2	1.13 (0.28, 4.52)	56	11.08 (8.51, 14.44)	47	11.56 (8.66, 15.42)
Meningitis aseptic	13	10.56 (6.12, 18.23)	28	5.10 (3.51, 7.41)	74	8.6 (6.82, 10.85)			6	5.33 (2.39, 11.89)	18	5.53 (3.48, 8.80)	55	21.52 (16.45, 28.15)
Meningitis noninfective					3	22.53 (6.86, 73.96)								
Meningoradiculitis	3	109.68 (34.02, 353.62)	3	24.43 (7.58, 78.75)										
Pachymeningitis			5	14.01 (5.73, 34.25)	6	10.54 (4.64, 23.91)							2	11.55 (2.86, 46.68)
Total	29		79		156		2		8		79		104	

PT, preferred term.

## 4 Discussion

To our knowledge, this constitutes the largest pharmacovigilance characterization of ICIs-associated aseptic meningitis (SMQ: 20000134), leveraging FAERS data to identify significant safety signals for five ICI monotherapies and ipilimumab/nivolumab combination therapy. Through integrated disproportionality and Bayesian analyses, our study establishes the most comprehensive post-marketing safety profile of this neurological irAE to date.

From 2011 to 2024, 498 cases of ICIs-associated AM were documented in FAERS. Pharmacovigilance analysis identified pembrolizumab and ipilimumab/nivolumab combination therapy as predominant suspected agents. Patients presented at median 64 years (range: 20-90), consistent with prior reports of drug-induced meningitis (median 56 years, range: 19-82) (10). In our cohort, males comprised 46.2% of reported cases, aligning with prior studies where male prevalence ranged from 52% to 65% (10, 12), where head/neck cancers (24.7%) and hematolymphoid malignancies (16.1%) constituted the primary indications. This distribution contrasts with Nannini et al.’s case series, which reported melanoma (52.5%, n=21), lung cancer (25.0%, n=10), and renal cell carcinoma (15.0%, n=6) as predominant malignancies (10). Notably, 98% of cases met serious adverse event criteria, with 11.4% fatalities underscoring the life-threatening nature of ICIs-associated AM. This mortality rate substantially exceeds the 7.41% reported for AM in the Japanese Adverse Drug Event Report database (13), highlighting the critical severity profile observed in our cohort. Ethnic differences and indication-specific variations may drive this divergence: whereas the Japanese cohort primarily received ICIs for non-small cell lung cancer (13), our population included higher-risk indications like head/neck cancers and hematolymphoid malignancies.

The time-to-onset window for ICIs-associated AM spans broadly but typically occurs within weeks to months of therapy initiation, though rare manifestations may emerge after 14 treatment cycles (10). The median time-to-onset of ICIs-associated AM was 34 days (range: 0-2194) in FAERS reports. This aligns with the landmark case series reporting 2 treatment cycles (median; range 1-14) (10), corresponding to approximately 28 days for PD-L1 inhibitors (avelumab, atezolizumab and durvalumab) and 42 days for CTLA-4-containing regimens (ipilimumab monotherapy and combination) and PD-1 inhibitors (pembrolizumab and cemiplimab). In patients treated with atezolizumab, AM manifested at a median onset of 10 days (range: 0-730,n=38)–earlier than observed with other ICIs except durvalumab (n=2 limited cases). This accelerated neurotoxicity profile aligns with pivotal NSCLC trials (14), case series documenting AM onset within 11–14 days post-first cycle (10), and consistently observed encephalitis manifestations within 13–14 days of first dose across malignancies (15). Nivolumab-treated patients demonstrated a prolonged median onset of AM (median: 81 days; range: 0-730; n=33), exceeding the overall ICIs-associated AM timeline (34 days). While this contrasts with a smaller cohort reporting neurologic serious adverse events at median 48 days (range: 1-170; n=13), the broad onset window (0–730 days) encompasses both early and delayed presentations (16). Notably, an extreme case revealed 4-year continuous nivolumab exposure (480mg monthly) prior to AM manifestation (17). Moreover, our study confirmed significantly earlier onset of AM with ipilimumab/nivolumab combination therapy versus nivolumab monotherapy (32 days [range, 0-336] VS 81 days [range, 0-2194]). This finding aligns with prior observational studies reporting accelerated toxicity under dual checkpoint blockade, where median time to onset was 42 days (range, 5-131) versus 48 days (range, 1-170) for nivolumab monotherapy (16). Our analysis found that ipilimumab/nivolumab combination therapy was associated with earlier onset and stronger signals of AM than monotherapies. This likely reflects synergistic immune activation: CTLA-4 blockade enhances T-cell priming, while PD-1 inhibition sustains effector responses, together amplifying autoimmune reactions against neural antigens. Clinically, this results in faster onset of AM in combination regimens, underscoring the need for intensive monitoring during early treatment cycles (18).

Although our analysis revealed stronger signals in monotherapy compared to some combination regimens, the FAERS database lacks granular information on patient-level characteristics such as comorbidities, immune status, prior infections, and concomitant medications. These unmeasured confounders may substantially influence the risk of aseptic meningitis. Future prospective cohort studies and registry data are warranted to clarify the contribution of host-related factors to ICI-associated neurotoxicity.

Our pharmacovigilance analysis identified significant safety signals for AM associated with five ICI monotherapies (ipilimumab, nivolumab, pembrolizumab, atezolizumab, cemiplimab) and ipilimumab/nivolumab combination therapy in FAERS (ROR 95% CI >1 for all. Durvalumab and avelumab showed no significant associations, potentially attributable to their later market approval (2017) and consequently lower cumulative exposure compared to earlier-launched ICIs. This finding aligns with Sato et al.’s Japanese database study (April 2004–March 2019), which identified no AM cases linked to these agents (13). Moreover, the detected signal pattern is corroborated by global pharmacovigilance data: VigiBase data confirmed ICIs-related non-infectious meningitis signals (ROR 3.1, 95% CI[2.5, 3.9]) (8), while Sato et al. reported similar findings in Japanese databases (ROR 1.79, 95% CI[1.17, 2.62]) (13). Furthermore, of all ICI monotherapies, ipilimumab had the strongest correlation with AM, and the underlying mechanism for this remains to be discovered (19).

The established clinical benefits of combining anti-CTLA-4 and anti-PD-1/PD-L1 agents have established dual immune checkpoint blockade as a therapeutic standard for multiple malignancies. However, this regimen is associated with increased multisystem toxicities, with nearly one-third of fatal irAEs attributed to pneumonitis (35%), hepatitis (22%), colitis (17%), neurological events (15%), and myocarditis (8%) (3). Our pharmacovigilance analysis further corroborates this risk profile, revealing significantly stronger safety signals for ipilimumab/nivolumab combination therapy versus nivolumab monotherapy (ROR 2.64, 95% CI 1.98–3.51)–mirroring Vigibase findings (ROR 2.7, 95% CI 1.5–4.7) (ROR 2.7, 95% CI 1.5–4.7) (8). These findings align with meta-analyses demonstrating elevated rates of all-grade and high-grade irAEs (e.g., pruritus, rash, diarrhea, colitis, ALT elevation, pneumonitis; n=2,946) (20), underscoring the necessity for standardized pharmacological prophylaxis (e.g., corticosteroid premedication) in patients receiving anti-CTLA-4/PD-1 combinations for metastatic tumors.

The pathophysiology of ICIs-induced neurological toxicities involves multifaceted immune dysregulation, primarily driven by checkpoint blockade (e.g., CTLA-4, PD-1/PD-L1) potentiating T-cell activation against neural antigens. Molecular mimicry explains organ-specific heterogeneity, such as shared ganglioside expression between melanoma cells and Schwann cells—accounting for elevated neurotoxicity in melanoma cohorts (3, 21, 22). Humoral mechanisms significantly contribute, with neuromuscular/brain-reactive autoantibodies (e.g., anti-GAD65, anti-AChR) detected in 68% of neurological irAE patients versus 12% in unaffected individuals, implicating antibody-mediated neural injury (21, 24). Concurrently, activated cytotoxic T cells infiltrate neural tissues—evidenced by CSF lymphocytosis in encephalitis and clonal T-cell expansion in ICIs-myositis biopsies—disrupting the blood-brain/blood-nerve barriers and amplifying cytokine release (e.g., IFN-γ, TNF-α) (22). This process is exacerbated by compromised anatomical protections: ICIs blockade counteracts PD-L1 upregulation on astrocytes/microglia during neuroinflammation, while shared T-cell clones targeting antigens (e.g., α-myosin) in tumors and neural tissues amplify cross-organ damage (23).

Our pharmacovigilance analysis confirms that AM represents a clinically significant neurological irAE in patients receiving ICIs. Clinicians must recognize that this toxicity may progress to fatal complications such as encephalitis or acute disseminated encephalomyelitis. Early diagnosis through enhanced neuroimaging (e.g., leptomeningeal enhancement on MRI) and CSF analysis is critical, as prompt intervention with high-dose corticosteroids can mitigate severe outcomes in >90% of cases (9). Spontaneous reporting systems, such as VigiBase and FAERS, are vital for detecting rare safety signals. Recognizing the specific ICIs regimen-associated risk profiles and features of AM is crucial. Furthermore, heightened awareness of this potential adverse event among oncologists, emergency physicians, clinical pharmacists, and other relevant specialists is imperative. These findings warrant consideration for optimizing clinical decision-making regarding ICIs therapy and informing the design of future clinical trials evaluating different ICI regimens.

Our data indicate that over 70% of cases occurred within three months of therapy initiation, highlighting a critical window for intensive monitoring. Beyond this period, mitigation strategies remain less well defined. Potential approaches may include periodic neurological assessment, early neuroimaging and CSF evaluation when nonspecific symptoms emerge, and judicious dose modification or temporary discontinuation in patients with high-risk profiles. Future research should evaluate whether tailored prophylactic immunosuppression or biomarker-guided surveillance can further mitigate risk in the later phases of therapy.

Several methodological constraints require acknowledgment. First, inherent limitations of FAERS—such as underreporting, incomplete documentation, and selective reporting—may introduce selection bias. Although duplicate cases were removed and standardized definitions applied, residual bias remains. Prospective approaches, including registry-based cohorts and active surveillance, are needed to better control for such bias. Second, FAERS does not provide denominator data, making it impossible to calculate incidence rates or absolute risks. Therefore, our findings indicate disproportionality signals rather than true incidence. Third, the database’s qualitative nature precludes quantification of AM incidence rates, as neither total adverse reaction counts nor patient exposure denominators are systematically captured. Finally, while detected signals indicate statistical associations between ICIs and AM, they cannot establish biological causation without prospective validation. Importantly, despite these pharmacovigilance system constraints, FAERS effectively characterizes key aspects of ICIs-associated AM including temporal patterns, clinical spectra, and manifestation profiles, thereby generating testable hypotheses for future clinical studies.

## 5 Conclusion

In conclusion, our FAERS database analysis identifies a disproportionate reporting signal for AM associated with ICI monotherapies (ipilimumab, nivolumab, pembrolizumab, cemiplimab, atezolizumab) and ipilimumab/nivolumab combination therapy. This signal was particularly pronounced with ipilimumab-containing regimens, either as monotherapy or in combination with nivolumab. These findings warrant heightened clinical vigilance for this potentially serious irAE. Further pharmacovigilance investigations, prospective cohort analyses, and dedicated clinical trials are needed to elucidate the underlying mechanisms and develop evidence-based management strategies for ICIs-associated AM.

## Data Availability

The original contributions presented in the study are included in the article/supplementary material. Further inquiries can be directed to the corresponding author.
